# Serial Testing for Tuberculosis: Can We Make Sense of T Cell Assay Conversions and Reversions?

**DOI:** 10.1371/journal.pmed.0040208

**Published:** 2007-06-12

**Authors:** Madhukar Pai, Richard O'Brien

## Abstract

The authors discuss a new study, which they say is a valuable addition to the existing literature on the performance of interferon gamma release assays in serial testing for TB infection.

## Serial Testing for Tuberculosis

In many high-income countries with low rates of tuberculosis (TB), serial testing for latent TB infection (LTBI) is recommended for persons at increased risk of TB exposure. For example, periodic screening of health care workers for LTBI is an important component of nosocomial TB infection control programs. Serial testing is also performed during household contact investigations. However, the conventional tuberculin skin test (TST) has known limitations in accuracy and reliability [1,2]. Furthermore, the interpretation of serial TST results is particularly complicated because of non-specific variations in test results, boosting, conversions, and reversions (see Glossary for definitions) [3]. In this context, the development of more specific, in vitro assays for LTBI—interferon gamma (IFNγ) release assays (IGRAs)—is a welcome development. These assays are highly specific, especially in bacillus Calmette Guérin (BCG)-vaccinated populations [4,5].

## Use of T Cell Assays for Serial Testing

In theory, IGRAs have features that make them ideal for serial testing. They are more specific than TST, can be repeated any number of times without sensitization and boosting, the testing protocol requires only one visit, and unlike the TST, there is no need for a baseline two-step testing protocol to avoid misclassifying TST increases due to boosting as true conversions due to new infection. Furthermore, there is some evidence, although not consistent, that IGRAs may be better at detecting recent rather than remote infection [6].

**Linked Research Article**

This Perspective discusses the following new study published in *PLoS Medicine*:

Hill PC, Brookes RH, Fox A, Jackson-Sillah D, Jeffries DJ, et al. (2007) Longitudinal assessment of an ELISPOT test for Mycobacterium tuberculosis infection. PLoS Med 4(6): e192. doi:10.1371/journal.pmed.0040192

Philip Hill and colleagues report that both ELISPOT conversion and reversion occur after M. tuberculosis exposure in an endemic country and that the ELISPOT tests results agree poorly with results from the tuberculin skin test.

Are these theoretical advantages borne out in real-life studies? Until recently, longitudinal data were lacking on performance of IGRAs in serial testing. In this context, the new study by Philip Hill and colleagues, published in *PLoS Medicine* [7], is a valuable addition to the existing literature on IGRA performance in serial testing [8–11]. By serially testing a fairly large number of exposed contacts in a high-prevalence country (i.e., The Gambia), the authors have shown that IGRA, specifically ELISPOT, conversions and reversions frequently occur after Mycobacterium tuberculosis exposure.

In order to interpret and use IGRAs in serial testing, we need evidence on several key questions [4,6,12]: (1) What is the reproducibility of T cell responses over time (within-subject variations over time)? (2) What is an IGRA “reversion,” and what threshold should be used to define reversion? What is the clinical significance and prognosis of an IGRA reversion? (3) What is an IGRA “conversion,” and what threshold (cut-off) should be used to define conversion? How can IGRA conversions be distinguished from nonspecific (random) variations in T cell responses over time? (4) What is the prognosis of an IGRA conversion? Are individuals with strong conversions (i.e., large increases in IFNγ responses over time) at higher risk of progression to active disease than individuals with weak conversions or negative results? (5) Will treatment of individuals with IGRA conversions reduce their risk of progression to active disease?

Unfortunately, none of the published studies provide strong evidence on any of these and other key questions (Box 1). For example, there are virtually no data on the reproducibility of IGRAs, particularly with regard to within-subject variability of T cell responses during serial testing [6]. There are limited data on how much IFNγ responses will increase following new infection and how to differentiate this increase from changes due to test-related error or nonspecific biological variations over time. Without such data, the results of serial IGRA testing will be difficult to interpret [6].

**Box 1. Outstanding Research Questions for Longitudinal and Serial Testing Studies**

What is the amount of random, biological variability of IFNγ responses over time, within the same individuals, including day-to-day, week-to-week, and month-to-month variability of IFNγ levels in the absence of TB exposure?

For serial testing with IGRAs, which threshold for IFNγ (cut-off point) is optimal for distinguishing between true infection (i.e., conversion) and nonspecific, random variation?

How should an IGRA reversion be defined, how commonly do reversions occur, and what is the clinical/epidemiological significance of reversions? What factors are associated with IGRA reversions, including treatment, baseline IFNγ levels, and variability around cut-off points?

Among individuals with positive IGRA results, are those with strong and/or rising IFNγ responses at greater risk for progressing to active disease? Is it possible to identify an IGRA cut-off point that is predictive of incipient or subclinical TB disease?

Among those screened with serial TST and IGRA, what is the concordance between IGRA and TST conversions? What is the correlation between changes in absolute TST reactions and IFNγ levels? Will treatment of IGRA-positive subjects (or IGRA conversions) reduce the future probability of active TB?

Adapted with permission from reference [6].

Existing studies, including the study by Hill and colleagues, clearly demonstrate that conversions, reversions, and nonspecific variations occur with IGRA serial testing, just as they do with TST serial testing (Table 1) [7–11]. Serial testing studies show that IGRAs are highly dynamic tests and that T cell responses, especially weakly positive responses, tend to fluctuate over time, even in the absence of specific treatment [7–11]. Although limited, available data suggest that positive IGRA results vary more than negative results. This variation is expected, because positive results have room to vary on both sides, while negative results are constrained to vary between zero and the diagnostic cut-off (which is set low in commercially available assays). Overall, IGRAs may be inherently prone to conversions and reversions, and this dynamic characteristic raises the concern that these assays may be too labile or unstable to be used in serial testing. Many of the published serial testing studies have been done in high-incidence countries. It is unclear whether similar findings will be seen in countries where exposure to TB and other nontuberculous mycobacteria is less frequent. Clearly, we have yet to fully understand the various forces that drive or modulate T cell responses.

**Table 1:**
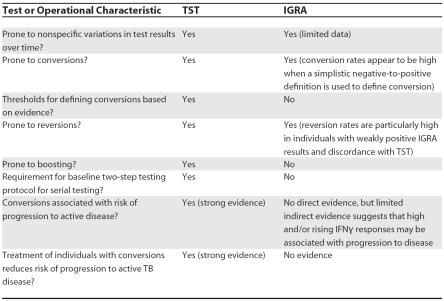
Comparison of TST and IGRA with Respect to Various Serial Testing Characteristics

## IGRA Reversions and Their Prognosis

An interesting finding in the Gambian study is the high rate of reversions among household contacts, even within a time span of three months and despite the newness of exposure [7]. Previous studies have also found high reversion rates with IGRAs [8,9,11]. In general, reversions are least likely when baseline IFNγ responses are strong and concordantly positive (i.e., positive by both TST and IGRA). Concordantly positive individuals are likely to have very strong IFNγ responses. Consequently, unless their responses drop dramatically, reversions are not likely. In contrast, IGRA reversions are more likely when the baseline test results are discordant (i.e., positive by IGRA but negative by TST) [7–9,11]. Discordant results are often weakly positive, and weakly positive IFNγ levels are likely to be just above the diagnostic threshold. Therefore, even minor nonspecific variations around the threshold can lead to apparent reversions.

Why do IGRA reversions occur? Some reversions may reflect clearing of TB infection (spontaneous or due to treatment). Some reversions may merely be due to biological variations among IGRA positive individuals, and some reversions may be due to variability in laboratory and test procedures. Hill and colleagues suggest that IGRA responses are inherently transient and generally require continued exposure to TB antigens to maintain high frequencies [7]. They argue that reversions may simply reflect the life cycle of M. tuberculosis, where the mycobacterium enters a dormant state in which it may not reliably secrete antigens such as early secreted antigen target 6 (ESAT-6) and culture filtrate protein 10 (CFP-10), but instead secrete other antigens (which are not used in currently available IGRAs) [7]. Alternatively, ESAT-6 and CFP-10 may be secreted intermittently over the life cycle of M. tuberculosis, which may partly explain the variations over time [7]. These hypotheses are worthy of further study.

**Glossary of Key Terms**

**Boosting of TST:** Boosting of TST upon retesting in the absence of new infection is immunologic recall of pre-existing hypersensitivity to TB (i.e., recall of waned immunity).

**Conversion:** Conversion is the development of new immunological reactivity (i.e., increase in TST or IFNγ responses) following new infection with M. tuberculosis.

**Reversion:** A previously positive TST or IGRA result becomes negative upon repeat testing (i.e., decrease in TST or IFNγ responses).

**Threshold for diagnosis of TB infection:** Cut-off used to detect existing latent TB infection with a one time screening. With TST, cut-offs vary according to risk (e.g., 5, 10, and 15 mm). With IGRA, the cut-offs depend on the type of assay and varies between commercial and in-house assays.

**Threshold for conversion:** Cut-off used to detect new TB infection during serial testing. With TST, conversion is usually defined as a second TST induration of at least 10+ mm and an increase of ≥6 mm (some guidelines suggest an increase of ≥10 mm). With IGRA, the commonly used definition is a change from negative to positive, but this simplistic definition does not specify the amount of increase over the baseline IFNγ level.

**Discordance between TST and IGRA:** Disagreement between the two test results; for example, TST is positive but IGRA is negative.

In routine practice, IGRA-positive individuals will not undergo repeated testing (just as TST positive individuals do not usually get retested). In that case, do reversions matter outside the setting of research? Probably not, until we learn more about the significance and prognosis of IGRA reversions. Do IGRA reversions indicate clearance or resolution of TB infection? Are individuals with reversions susceptible to new infections? Unfortunately, our existing knowledge base is insufficient to answer these questions. Cohort studies are needed to define the prognosis of IGRA reversions (and conversions). Such studies are ongoing and will help to settle unanswered questions [13].

## IGRA Conversions and Their Significance

Although a fairly high rate of IGRA conversions has been documented in high-risk populations in highly endemic settings [8,11], there is no consensus on how to define and interpret conversions. Data are lacking on key questions, such as: How much increase in IFNγ indicates a true new infection, and how much is merely test or biological variability? Should the same threshold be used for diagnosis of LTBI as well as conversions (see Box 1)? If not, what is the ideal data-driven approach to deriving cut-offs for conversions?

Some studies show that if a simplistic negative-to-positive cut-off is used to define conversions, conversion rates may be higher with IGRAs than with TSTs [8,11]. This higher conversion rate could indicate higher sensitivity for conversions (not necessarily for diagnosis of LTBIs). However, some proportion of this higher conversion rate is probably due to minor nonspecific variations around the diagnostic cut-off. For example, if the ELISPOT spot count increased from just below the cut-off to a level just above, is that a real conversion? A more stringent cut-off for conversion may help avoid misclassifying minor variations as conversions [8]. The use of a stringent cut-off for conversion would be analogous to the TST threshold for conversion, which requires an increase of at least 6 mm of induration above the baseline value to an induration of at least 10 mm [3].

None of the existing studies, including the study by Hill and coworkers, have used data-derived cut-offs based on amount of variation in T cell responses over time, an important area for future research (Box 1). Until additional data become available, health professionals who interpret serial IGRA testing must learn to critically interpret continuous IFNγ values, and not merely rely on dichotomous (positive/negative) results. To facilitate such interpretation, laboratories should report continuous as well as dichotomous results.

## Prognosis of IGRA Conversions and Potential Use of IGRAs as a Predictive Test

With the TST, the risk of development of active TB has been established in several cohort studies (reviewed in [1]). Also, from controlled clinical trials, we know that treatment of TST-positive persons reduces the risk of active disease [14]. This knowledge has resulted in guidelines for targeted TST testing and treatment for LTBI [14]. Unfortunately, there are no equivalent data for IGRAs. Although the data are limited to one small study [15], of an association between strong IFNγ response to ESAT-6 and subsequent progression to active TB among household contacts of index cases, the prognosis of a positive IGRA result has yet to be determined (Box 1).

What is the prognosis of an IGRA conversion? Conversion implies recent infection and the prognosis for recent (incident) infection is expected to differ from the prognosis of already existing (prevalent) infection. Furthermore, the prognosis of a “strong conversion” may well be different from that of a “weak conversion.” There are no data that directly answer the question of prognosis of conversions, but emerging data suggest that responses to both ESAT-6 and CFP-10 correlate closely with bacterial replication in vivo and with progression from infection to disease [6,13].

**Figure 1:**
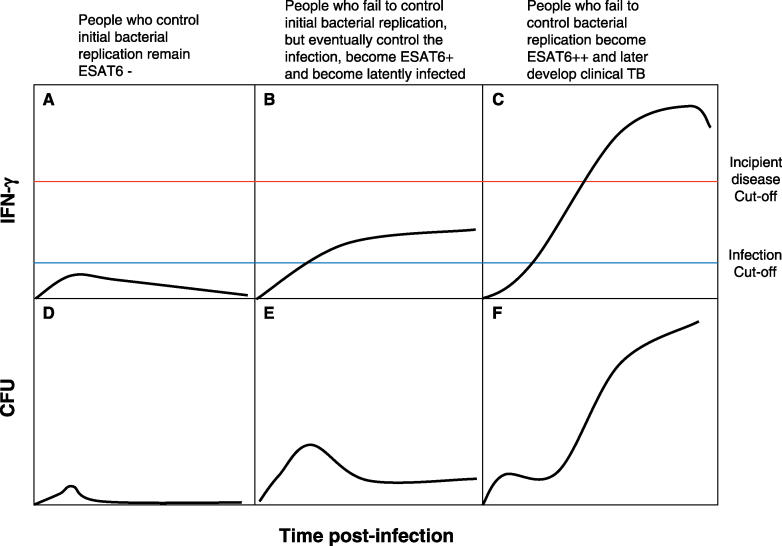
A Schematic of the Postulated Correlation between Bacterial Load, T Cell Response, and Clinical Outcome Initial infection might be controlled at its onset with minimal bacterial replication (measured as colony-forming units [CFU]) and induction of ESAT-6 responses (A and D). In such cases, the T cell response may be below the diagnostic cut-off. However, in most cases, initial bacterial replication reaches a point at which it induces an ESAT-6-specific IFNγ response to increase above the established cut-off value (infection threshold), enabling the diagnosis of an individual as latently infected (B and E). In most cases, individuals control the infection, resulting in latent infection, but some develop active TB disease associated with progressive bacterial replication. This is accompanied by increasing and strong ESAT-6 responses and, as hypothesized here, an incipient (higher) disease cut-off value may predict subsequent development of progressive disease (C and F). Reproduced with permission from [13].

Recently, Andersen and colleagues hypothesized that high and/or rising levels of IFNγ produced in response to ESAT-6 by T cells from recently TB-infected individuals may signal incipient disease and thus might serve as a prognostic marker for subsequent development of overt clinical disease in the near future (Figure 1) [13]. They reviewed animal and human studies that provide preliminary evidence that individuals with recent exposure have vigorous increases in T cell responses, probably due to active bacterial replication [13]. Because it is well documented that individuals with recent TST conversions have a high likelihood of progressing to active disease, it is plausible that strong increases in IFNγ responses after recent exposure might predict progression to active disease. Thus, instead of using IGRAs only as dichotomous tests, the actual IFNγ level or rising levels (i.e., conversion) may allow the identification of a subgroup among infected individuals who may be in the process of developing active TB. If confirmed in large-scale cohort studies, this approach may allow for more targeted treatment to prevent active disease among latently infected individuals.

## Abbreviations

CFP-10: culture filtrate protein 10
ESAT-6: early secreted antigen target 6
IFNγ: interferon gamma
IGRA: interferon gamma release assay
LTBI: latent tuberculosis infection
TB: tuberculosis
TST: tuberculin skin test
